# Supplementary material for: Antimicrobial Stewardship during COVID-19 Outbreak: A Retrospective Analysis of Antibiotic Prescriptions in the ICU across COVID-19 Waves
Source: Antibiotics (Basel). 2022 Oct 30;11(11):1517. doi: 10.3390/antibiotics11111517 (PMC9686868; doi:10.3390/antibiotics11111517)
Supplement: Supplementary file 1 [file antibiotics-11-01517-s001.zip › antibiotics-1921952-supplementary.pdf]

Table S1. Definition for suspected or proven infections (based on international definitions)

Site	Criteria for diagnosis
**Ventilator-associated pneumonia**	Evolving radiological lung opacity AND identification of micro-organism in:<br>1. Tracheal aspirates through endotracheal tube with at least 10^6 colonies forming unit/mL<br>2. Bronchial-alveolar lavage with at least 10^4 colonies forming unit/mL<br>3. Protected brush with at least 10^3 colonies forming unit/mL<br>4. Collection of abscess or pleural fluid
**Pneumonia**	Evolving radiological lung opacity AND fever (body temperature > 38°C) AND identification of micro-organism by cytobacteriological exam with:<br>1. < 10 epithelial cells<br>2. AND > 25 neutrophilic polynuclear<br>3. No more than one micro-organism identified with at least 10^7 colonies forming unit/mL
**Intra-abdominal infection**	Pus or abscess collected during surgery OR positive blood culture collected at the onset of disease
**Catheter-related infection**	Positive blood culture (collected by a venous puncture) AND one of the following criteria:<br>1. Local infection AND identification of the same micro-organism in pus and peripheral blood<br>2. Positive culture of catheter AND identification of the same micro-organism in peripheral blood
**Bacteremia**	At least one positive blood culture collected during fever episode excepted for negative coagulase *Staphylococcus*, *Bacillus sp*., *Corynebacterium sp*., *Propionibacterium sp*., *Micrococcus sp.* for which at least two positive blood cultures collected at different sites are required
**Tissue or skin infection**	Presence of a least two of the following signs:<br>1. Local pain<br>2. Tumefaction<br>3. Local heat<br>4. Sensibility<br>5. Redness<br>AND at least one of the following signs:<br>1. Positive culture of micro-organism collected at the suspected site<br>2. Micro-organism identified in blood culture
**Uro-genital infection**	Positive urine culture (no more than two bacteria) AND fever (body temperature > 38°C) without any other suspected infection site
**ENT infection**	Presence of at least two of the following criteria:<br>1. Local pain<br>2. Tumefaction<br>3. Local heat<br>4. Redness<br>5. Sensibility<br>6. Abscess or pus<br>AND assessment of local inflammation by either ultrasound or CT-scan

Table S2. Site of infection

Site	n	Frequency
**Lung**	158	88%
associated bacteremia	4	
**Bacteremia**	10	
**Ear, nose and throat (ENT)**	5	3%
cellulitis	2	
parotiditis	2	
sinusitis	1	
**Abdominal**	4	2%
**Urinary tract**	3	1.7%

Table S3. Bacterial identification

Microbiological identification	n	Frequency
Sterile	86	41.95%
S. aureus oxacillin-S	29	14.15%
P. aeruginosa ticarcillin-S	16	7.80%
Klebsiella spp cefotaxime-S	15	7.32%
H. influenzae ampicillin-S	6	2.93%
S. negative coagulase oxacillin-S	6	2.93%
Enterobacter spp cefotaxime-S	5	2.44%
S. negative coagulase oxacillin-R	5	2.44%
S. pneumoniae amoxicillin-S	5	2.44%
Klebsiella spp ESBL	4	1.95%
Corynebacteriae spp	3	1.46%
Enterococcus spp amoxicillin-S	3	1.46%
Morganella spp cefotaxime-S	3	1.46%
Hafnia spp cefotaxime-S	2	0.98%
Other *Streptococcus* spp amoxicillin-S	2	0.98%
S. aureus oxacillin-R	2	0.98%
S. maltophilia ticarcillin-R	2	0.98%
Citrobacter spp cefotaxime-S	1	0.49%
E. coli ESBL	1	0.49%
H. influenzae ampicillin-R	1	0.49%
M. catarrhalis ampicillin-S	1	0.49%
Other Gram positive cocci	1	0.49%
Other non *Enterobacteriae* Gram-negative bacilli	1	0.49%
Other *Pseudomonas* spp	1	0.49%
Other *Streptococcus* spp amoxicillin-R	1	0.49%
S. negative coagulase - without precision	1	0.49%
S. maltophilia ticarcillin-S	1	0.49%
S. pneumoniae - without precision	1	0.49%

ESBL : extended spectrum beta-lactamase

Table S4. Type of empirical antibiotic treatments used and their frequencies among other antibiotics and among the population of patients receiving empirical antibiotic treatment

Antibiotics	n	Frequency* (%)	Frequency in the population** (%)
Piperacillin/Tazobactam	95	47.5	67.4
Cefotaxime	35	17.5	24.8
Amikacin	25	12.5	17.7
Linezolid	18	9.0	12.8
Vancomycin	5	2.5	3.5
Imipenem-Cilastatin	4	2.0	2.8
Cefepime	3	1.5	2.1
Gentamicin	3	1.5	2.1
Meropenem	3	1.5	2.1
Levofloxacin	2	1.0	1.4
Amoxicillin- Clavulanate	1	0.5	0.7
Azithromycin	1	0.5	0.7
Aztreonam	1	0.5	0.7
Ciprofloxacin	1	0.5	0.7
Erythromycin	1	0.5	0.7
Metronidazole	1	0.5	0.7
Ofloxacin	1	0.5	0.7

*Frequency of the antibiotic among all the antibiotics used

**Frequency of the antibiotic in the population of patient (n=141)

Table S5: Frequency of bacteria detected according to the type of biological sample

Type of sample	Bacteria	Number	Frequency(%)
Bronchial aspirate	*S. aureus* methicillin-S	20	27.40%
Bronchial aspirate	*Klebsiella* cefotaxime-S	12	16.44%
Bronchial aspirate	*P. aeruginosa* ticarcillin-S	10	13.70%
Bronchial aspirate	*H. influenzae* ampicillin-S	6	8.22%
Bronchial aspirate	*Corynebacteriae* spp	3	4.11%
Bronchial aspirate	*Klebsiella* spp ESBL	3	4.11%
Bronchial aspirate	*S. pneumoniae* amoxicillin-S	3	4.11%
Bronchial aspirate	*Enterobacter* spp cefotaxime-S	2	2.74%
Bronchial aspirate	*S.* negative coagulase methicillin-R	2	2.74%
Bronchial aspirate	*E. coli* ESBL	1	1.37%
Bronchial aspirate	*Enterococcus* spp amoxicillin-S	1	1.37%
Bronchial aspirate	*H. influenzae* ampicillin-R	1	1.37%
Bronchial aspirate	*Hafnia* spp cefotaxime-S	1	1.37%
Bronchial aspirate	*M. catarrhalis* ampicillin-S	1	1.37%
Bronchial aspirate	*Morganella* spp cefotaxime-S	1	1.37%
Bronchial aspirate	Other Gram Positive Cocci	1	1.37%
Bronchial aspirate	Other non *Enterobacteriae* Gram-negative Bacilli	1	1.37%
Bronchial aspirate	Other *Pseudomonas*	1	1.37%
Bronchial aspirate	Other *Streptococcus* amoxicillin-S	1	1.37%
Bronchial aspirate	*S. maltophilia* ticarcillin-R	1	1.37%
Bronchial aspirate	*S. maltophilia* ticarcillin-S	1	1.37%
Bronchial aspirate + Hemoculture	*S. aureus* methicillin-S	1	100.00%
Bronchial aspirate + BAL	*S. aureus* methicillin-S	1	100.00%
Urine antigen	*S. pneumoniae* - without precision	1	100.00%
Sputum	*Klebsiella* cefotaxime-S	3	27.27%
Sputum	*P. aeruginosa* ticarcillin-S	3	27.27%
Sputum	*S. aureus* methicillin-S	2	18.18%
Sputum	*Citrobacter* cefotaxime-S	1	9.09%
Sputum	*Enterobacter* spp cefotaxime-S	1	9.09%
Sputum	*S. aureus* methicillin-R	1	9.09%
Sputum + Bronchial aspirate	*S. aureus* methicillin-S	1	100.00%
Urine	*Enterobacter* spp cefotaxime-S	1	50.00%
Urine	*P. aeruginosa* ticarcillin-S	1	50.00%

Type of sample	Bacteria	Number	Frequency(%)
Urine + Hemoculture	*Klebsiella* ESBL	1	100.00%
Hemoculture	*S.* negative coagulase methicillin-S	5	41.67%
Hemoculture	*S.* negative coagulase methicillin-R	3	25.00%
Hemoculture	*Enterococcus* spp amoxicillin-S	1	8.33%
Hemoculture	*P. aeruginosa* ticarcillin-S	1	8.33%
Hemoculture	*S. aureus* methicillin-S	1	8.33%
Hemoculture	*S.* negative coagulase - without precision	1	8.33%
BAL	*S. aureus* methicillin-S	3	20.00%
BAL	*Morganella* spp cefotaxime-S	2	13.33%
BAL	*S. pneumoniae* amoxicillin-S	2	13.33%
BAL	*Enterobacter* spp cefotaxime-S	1	6.67%
BAL	*Enterococcus* spp amoxicillin-S	1	6.67%
BAL	*Hafnia* spp cefotaxime-S	1	6.67%
BAL	*Other Streptococcus* amoxicillin-R	1	6.67%
BAL	*Other Streptococcus* amoxicillin-S	1	6.67%
BAL	*P. aeruginosa* ticarcillin-S	1	6.67%
BAL	*S. aureus* methicillin-R	1	6.67%
BAL	*S. maltophilia* ticarcillin-R	1	6.67%
Sinuses	*S.* negative coagulase methicillin-S	1	100.00%

BAL: Bronchio-alveolar lavage, *ESBL* : extended spectrum beta-lactamase

Table S6: Details of the antibiotic de-escalation situations in which the empirical antimicrobial agent was replaced by a narrower spectrum antibiotic

Empirical antibiotic 1	Empirical antibiotic 2	Empirical antibiotic 3	Targeted antibiotic 1	Targeted antibiotic 2
Cefotaxime	0	0	Amoxicillin	0
Cefotaxime	Azithromycin	Amikacin	Cloxacillin	0
Piperacillin tazobactam	Linezolid	0	Oxacillin	0
Cefotaxime	Levofloxacin	0	Amoxicillin	0
Piperacillin tazobactam	Amikacin	0	Cloxacillin	0
Piperacillin tazobactam	0	0	Cefazolin	0
Piperacillin tazobactam	Linezolid	Amikacin	Amoxicillin clavulanic acid	0
Piperacillin tazobactam	Linezolid	0	Cefepime	0
Imipenem-cisplatin	0	0	Piperacillin tazobactam	0
Cefotaxime	Linezolid	0	Amoxicillin clavulanic acid	Oxacillin
Piperacillin tazobactam	Linezolid	0	Amoxicillin	0
Piperacillin tazobactam	0	0	Oxacillin	0
Piperacillin tazobactam	Linezolid	0	Cloxacillin	0
Piperacillin tazobactam	0	0	Oxacillin	0
Piperacillin tazobactam	Linezolid	0	Oxacillin	0
Piperacillin tazobactam	0	0	Cefotaxime	0
Cefotaxime	0	0	Amoxicillin	0
Piperacillin tazobactam	0	0	Amoxicillin	0
Cefotaxime	0	0	Oxacillin	0
Piperacillin tazobactam	Linezolid	0	Oxacillin	0
Piperacillin tazobactam	Erythromycin	Amikacin	Amoxicillin	0
Meropenem	Gentamicin	0	Piperacillin tazobactam	0
Piperacillin tazobactam	0	0	Amoxicillin	0
Vancomycin	0	0	Oxacillin	Sulfamethoxazole trimethoprim
Cefotaxime	0	0	Oxacillin	0
Piperacillin tazobactam	Amikacin	0	Amoxicillin clavulanic acid	0
Piperacillin tazobactam	0	0	Cefotaxime	0
Cefotaxime	0	0	Amoxicillin clavulanic acid	0
Piperacillin tazobactam	0	0	Cefepime	0

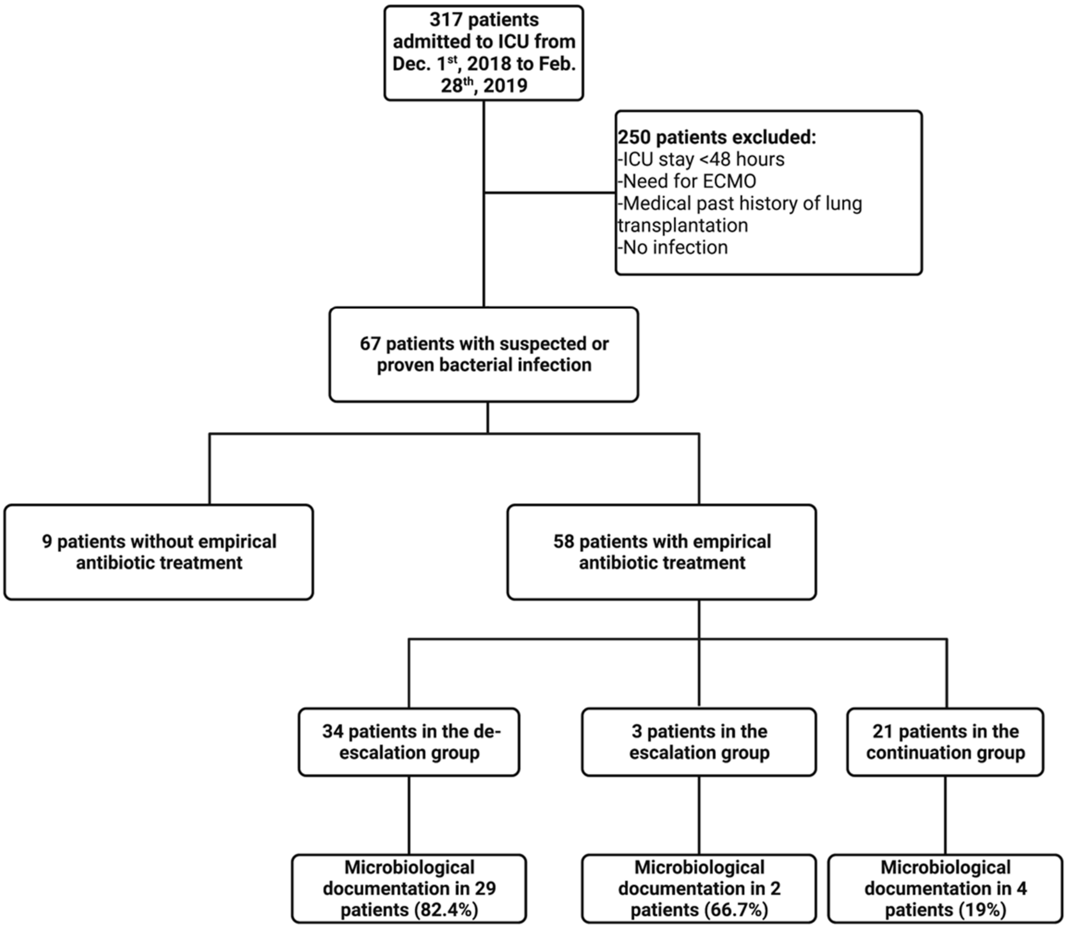

Figure S1: Flow chart of the control group

Table S7: Comparison of the COVID-19 and the control groups

	COVID-19 group (N=170)	Control group (N=67)	Overall (N=237)	P-value
**Age**				
Mean (SD)	63.3 (12.6)	60.6 (16.4)	62.5 (13.8)	0.363
Median [IQR]	66.0 [57.0 - 72.0]	64.0 [53.0 - 71.0]	65.0 [56.0 - 72.0]	
**Gender**				
F	46 (27.1%)	24 (35.8%)	70 (29.5%)	0.241
H	124 (72.9%)	43 (64.2%)	167 (70.5%)	
**Body Mass Index**				
Mean (SD)	30.0 (5.84)	24.5 (4.91)	28.8 (6.09)	0.001
Median [IQR]	29.0 [26.0 - 33.4]	24.0 [21.0 - 27.5]	28.0 [24.7 - 32.0]	
Missing	11 (6.5%)	20 (29.9%)	31 (13.1%)	
**Obesity**				
1	91 (53.5%)	56 (83.6%)	147 (62.0%)	0.001
2	71 (41.8%)	10 (14.9%)	81 (34.2%)	
Missing	8 (4.7%)	1 (1.5%)	9 (3.8%)	
**Immunosuppression***				
0	149 (87.6%)	55 (82.1%)	204 (86.1%)	0.366
1	21 (12.4%)	12 (17.9%)	33 (13.9%)	
**Invasive procedures as outpatients**				
0	169 (99.4%)	66 (98.5%)	235 (99.2%)	1
1	1 (0.6%)	1 (1.5%)	2 (0.8%)	
**Previous hospitalization < 90 days**				
0	151 (88.8%)	34 (50.7%)	185 (78.1%)	0.001
1	19 (11.2%)	33 (49.3%)	52 (21.9%)	
**Prior antibiotic course < 90 days**				
0	124 (72.9%)	36 (53.7%)	160 (67.5%)	0.00716

	COVID-19 group (N=170)	Control group (N=67)	Overall (N=237)	P-value
1	46 (27.1%)	31 (46.3%)	77 (32.5%)	
**Myocardial infarction**				
0	143 (84.1%)	57 (85.1%)	200 (84.4%)	1
1	27 (15.9%)	10 (14.9%)	37 (15.6%)	
**Congestive heart failure**				
0	165 (97.1%)	59 (88.1%)	224 (94.5%)	0.0154
1	5 (2.9%)	8 (11.9%)	13 (5.5%)	
**Vascular peripheral disease**				
0	161 (94.7%)	53 (79.1%)	214 (90.3%)	0.001
1	9 (5.3%)	14 (20.9%)	23 (9.7%)	
**Stroke**				
0	162 (95.3%)	62 (92.5%)	224 (94.5%)	0.601
1	8 (4.7%)	5 (7.5%)	13 (5.5%)	
**Dementia**				
0	168 (98.8%)	67 (100%)	235 (99.2%)	0.918
1	2 (1.2%)	0 (0%)	2 (0.8%)	
**Chronic respiratory failure**				
0	148 (87.1%)	55 (82.1%)	203 (85.7%)	0.437
1	22 (12.9%)	12 (17.9%)	34 (14.3%)	
**Connective tissue disease**				
0	168 (98.8%)	64 (95.5%)	232 (97.9%)	0.275
1	2 (1.2%)	3 (4.5%)	5 (2.1%)	
**Gastric ulcer**				
0	167 (98.2%)	62 (92.5%)	229 (96.6%)	0.0738
1	3 (1.8%)	5 (7.5%)	8 (3.4%)	
**Mild liver disease**				

	COVID-19 group (N=170)	Control group (N=67)	Overall (N=237)	P-value
0	166 (97.6%)	67 (100%)	233 (98.3%)	0.48
1	4 (2.4%)	0 (0%)	4 (1.7%)	
**Moderate to severe liver disease**				
0	168 (98.8%)	63 (94.0%)	231 (97.5%)	0.0976
1	2 (1.2%)	4 (6.0%)	6 (2.5%)	
**Kidney disease**				
0	160 (94.1%)	62 (92.5%)	222 (93.7%)	0.878
1	10 (5.9%)	5 (7.5%)	15 (6.3%)	
**Diabetes - without complications**				
0	118 (69.4%)	59 (88.1%)	177 (74.7%)	0.005
1	52 (30.6%)	8 (11.9%)	60 (25.3%)	
**Diabetes - with complications**				
0	164 (96.5%)	62 (92.5%)	226 (95.4%)	0.34
1	6 (3.5%)	5 (7.5%)	11 (4.6%)	
**Solid cancer without metastasis < 5 years**				
0	148 (87.1%)	54 (80.6%)	202 (85.2%)	0.289
1	22 (12.9%)	13 (19.4%)	35 (14.8%)	
**Solid cancer with metastasis**				
0	166 (97.6%)	62 (92.5%)	228 (96.2%)	0.14
1	4 (2.4%)	5 (7.5%)	9 (3.8%)	
**Leukemia**				
0	167 (98.2%)	66 (98.5%)	233 (98.3%)	1
1	3 (1.8%)	1 (1.5%)	4 (1.7%)	
**Lymphoma**				
0	167 (98.2%)	64 (95.5%)	231 (97.5%)	0.46

	COVID-19 group (N=170)	Control group (N=67)	Overall (N=237)	P-value
1	3 (1.8%)	3 (4.5%)	6 (2.5%)	
**Hypertension**				
0	86 (50.6%)	35 (52.2%)	121 (51.1%)	0.933
1	84 (49.4%)	32 (47.8%)	116 (48.9%)	
**Tobacco consumption**				
1	126 (74.1%)	53 (79.1%)	179 (75.5%)	0.408
2	44 (25.9%)	13 (19.4%)	57 (24.1%)	
Missing	0 (0%)	1 (1.5%)	1 (0.4%)	
**Charlson Score**				
Mean (SD)	4.60 (2.29)	5.36 (2.97)	4.81 (2.52)	0.0654
Median [IQR]	4.00 [3.00 - 6.00]	5.00 [3.00 - 7.00]	5.00 [3.00 - 6.00]	
**SAPS II at ICU admission**				
Mean (SD)	41.1 (15.7)	49.3 (16.4)	43.4 (16.3)	0.001
Median [IQR]	38.0 [29.0 - 49.0]	51.0 [36.0 - 60.0]	40.5 [30.8 - 53.0]	
Missing	0 (0%)	1 (1.5%)	1 (0.4%)	
**Acquired Resistance – Overall**				
0	161 (94.7%)	54 (80.6%)	215 (90.7%)	0.00942
1	9 (5.3%)	11 (16.4%)	20 (8.4%)	
Missing	0 (0%)	2 (3.0%)	2 (0.8%)	
**Acquired resistance in rectal swabs**				
0	167 (98.2%)	60 (89.6%)	227 (95.8%)	0.0659
1	3 (1.8%)	5 (7.5%)	8 (3.4%)	
Missing	0 (0%)	2 (3.0%)	2 (0.8%)	
**Acquired resistance in other samples**				
0	162 (95.3%)	54 (80.6%)	216 (91.1%)	0.005
1	8 (4.7%)	11 (16.4%)	19 (8.0%)	

	COVID-19 group (N=170)	Control group (N=67)	Overall (N=237)	P-value
Missing	0 (0%)	2 (3.0%)	2 (0.8%)	
**Therapeutic changes in antibiotics**				
De-escalation	47 (27.6%)	35 (52.2%)	82 (34.6%)	0.001
Incrementation	5 (2.9%)	3 (4.5%)	8 (3.4%)	
No change	118 (69.4%)	29 (43.3%)	147 (62.0%)	
**Total duration of antibiotic treatment (d)**				
Mean (SD)	8.86 (28.5)	41.1 (189)	18.0 (104)	0.0813
Median [IQR]	6.00 [3.00 - 7.00]	7.00 [4.00 - 11.0]	6.00 [3.00 - 8.00]	
**Relapse of infection**				
0	155 (91.2%)	60 (89.6%)	215 (90.7%)	0.785
1	14 (8.2%)	7 (10.4%)	21 (8.9%)	
Missing	1 (0.6%)	0 (0%)	1 (0.4%)	
**Recurrence of infection**				
0	143 (84.1%)	59 (88.1%)	202 (85.2%)	0.636
1	26 (15.3%)	8 (11.9%)	34 (14.3%)	
Missing	1 (0.6%)	0 (0%)	1 (0.4%)	
**Mechanical ventilation (d)**				
Mean (SD)	21.9 (16.5)	38.1 (202)	25.9 (101)	0.001
Median [IQR]	17.0 [11.0 - 30.0]	7.50 [3.00 - 15.5]	14.0 [8.00 - 26.0]	
Missing	13 (7.6%)	15 (22.4%)	28 (11.8%)	
**ICU length of stay (d)**				
Mean (SD)	27.7 (19.7)	13.8 (13.4)	23.8 (19.2)	0.001
Median [IQR]	21.0 [15.0 - 35.5]	9.00 [4.00 - 18.0]	18.0 [11.0 - 29.0]	
Missing	0 (0%)	2 (3.0%)	2 (0.8%)	
**Hospital length of stay (d)**				
Mean (SD)	35.1 (21.3)	31.5 (26.8)	34.1 (22.9)	0.0391

	COVID-19 group (N=170)	Control group (N=67)	Overall (N=237)	P-value
Median [IQR]	29.5 [20.0 - 46.0]	25.0 [11.0 - 40.5]	29.0 [18.0 - 45.0]	
Missing	0 (0%)	3 (4.5%)	3 (1.3%)	
**Sepsis to ICU discharge (d)**				
Mean (SD)	28.1 (20.3)	11.1 (8.16)	26.1 (20.1)	0.001
Median [IQR]	21.0 [15.0 - 35.5]	8.50 [5.00 - 16.3]	20.0 [12.8 - 32.0]	
Missing	0 (0%)	45 (67.2%)	45 (19.0%)	
**Sepsis to hospital discharge (d)**				
Mean (SD)	34.7 (23.7)	20.5 (14.6)	33.1 (23.3)	0.00335
Median [IQR]	29.0 [19.0 - 43.8]	16.0 [8.00 - 32.0]	28.0 [17.0 - 42.5]	
Missing	0 (0%)	46 (68.7%)	46 (19.4%)	
**Limitation of therapeutics (d)**				
0	156 (91.8%)	57 (85.1%)	213 (89.9%)	0.194
1	14 (8.2%)	10 (14.9%)	24 (10.1%)	
**ICU mortality**				
0	124 (72.9%)	51 (76.1%)	175 (73.8%)	0.736
1	46 (27.1%)	16 (23.9%)	62 (26.2%)	
**In-Hospital mortality**				
0	123 (72.4%)	49 (73.1%)	172 (72.6%)	1
1	47 (27.6%)	18 (26.9%)	65 (27.4%)	
**Day 28 Mortality**				
0	124 (72.9%)	50 (74.6%)	174 (73.4%)	0.919
1	46 (27.1%)	17 (25.4%)	63 (26.6%)	
**Day 90 Mortality**				
0	123 (72.4%)	47 (70.1%)	170 (71.7%)	0.858
1	47 (27.6%)	20 (29.9%)	67 (28.3%)	

SD: standard deviation, *IQR:* interquartile range, *BMI*: body mass index, *ICU*: intensive care unit, *MDR*: multidrug resistant, *SAPS II*: Simplified Acute Physiology Score, *AIDS:* acquired immunodeficiency syndrome, *(d):* days

Charlson score: age > 40 years, myocardial infarction, congestive heart failure, vascular peripheral disease, stroke, dementia, respiratory insufficiency, rheumatologic disease, gastric ulcer, liver disease, kidney disease, diabetes, central palsy, solid cancer with or without metastasis, leukemia, lymphoma, AIDS

*Immunosuppression: treatment with corticosteroid, chemotherapy, immunotherapy, or other immunosuppressive drugs, history of transplant, blood disease resulting in immunosuppression

** Overall acquired resistance was defined as the appearance of a MDR bacteria either in rectal swabs or other samples.
